# PKA and ERK, but not PKC, in the amygdala contribute to pain-related synaptic plasticity and behavior

**DOI:** 10.1186/1744-8069-4-26

**Published:** 2008-07-16

**Authors:** Yu Fu, Jeong Han, Titilope Ishola, Michelle Scerbo, Hita Adwanikar, Cara Ramsey, Volker Neugebauer

**Affiliations:** 1 Department of Neuroscience & Cell Biology, The University of Texas Medical Branch, Galveston, TX, 77555-1069, USA

## Abstract

The laterocapsular division of the central nucleus of the amygdala (CeLC) has emerged as an important site of pain-related plasticity and pain modulation. Glutamate and neuropeptide receptors in the CeLC contribute to synaptic and behavioral changes in the arthritis pain model, but the intracellular signaling pathways remain to be determined. This study addressed the role of PKA, PKC, and ERK in the CeLC. Adult male Sprague-Dawley rats were used in all experiments. Whole-cell patch-clamp recordings of CeLC neurons were made in brain slices from normal rats and from rats with a kaolin/carrageenan-induced monoarthritis in the knee (6 h postinduction). Membrane-permeable inhibitors of PKA (KT5720, 1 μM; cAMPS-Rp, 10 μM) and ERK (U0126, 1 μM) activation inhibited synaptic plasticity in slices from arthritic rats but had no effect on normal transmission in control slices. A PKC inhibitor (GF109203x, 1 μM) and an inactive structural analogue of U0126 (U0124, 1 μM) had no effect. The NMDA receptor-mediated synaptic component was inhibited by KT5720 or U0126; their combined application had additive effects. U0126 did not inhibit synaptic facilitation by forskolin-induced PKA-activation. Administration of KT5720 (100 μM, concentration in microdialysis probe) or U0126 (100 μM) into the CeLC, but not striatum (placement control), inhibited audible and ultrasonic vocalizations and spinal reflexes of arthritic rats but had no effect in normal animals. GF109203x (100 μM) and U0124 (100 μM) did not affect pain behavior. The data suggest that in the amygdala PKA and ERK, but not PKC, contribute to pain-related synaptic facilitation and behavior by increasing NMDA receptor function through independent signaling pathways.

## Introduction

The present study focused on the role of intracellular signaling mechanisms in the amygdala in pain-related plasticity and behavior. The amygdala is now recognized as an important player in the emotional-affective dimension of pain [1-9]. The laterocapsular division of the central nucleus of the amygdala (CeLC) is of particular importance, because it receives nociceptive ("pain-related") information directly from spinal cord and brainstem and indirectly, through the lateral-basolateral amygdala circuitry, from thalamus and cortex [1,8]. Our previous studies demonstrated central sensitization [10-15] and synaptic plasticity [10,16-19] in the CeLC in the kaolin/carrageenan-induced arthritis pain model. Recent imaging data also showed increased amygdala activation related to knee pain in patients with osteoarthritis [20]. Pain-related synaptic plasticity in the CeLC was confirmed in a model of chronic neuropathic pain [3] and was mimicked by tetanic stimulation of presumed nociceptive inputs from the brainstem [21].

A consequence of pain-related amygdala activation is increased pain behavior. Deactivation of the central nucleus decreased nocifensive and affective behavior associated with arthritic [9,10,22], formalin-induced [[2]; but see Tanimoto et al., 2003], visceral [23-25], and neuropathic pain [4]. However, the amygdala is also important for pain inhibition, particularly in the context of stress-induced and conditioned forms of analgesia [26-32]. The conditions under which the amygdala assumes pro- or anti-nociceptive functions and the underlying mechanisms remain to be determined.

Arthritis pain-related synaptic plasticity and central sensitization in the CeLC require the upregulation of presynaptic metabotropic glutamate receptors [12,16] and increased postsynaptic NMDA receptor function through a mechanism that involves NR1 phosphorylation by PKA [13,17]. Pain-related PKA activation in the CeLC appears to occur downstream of calcitonin gene-related peptide receptor CGRP1 [10] and corticotropin-releasing factor receptor CRF1[11,33]. Protein kinases such as PKA, PKC, and ERK, play important roles in the central sensitization of spinal cord neurons [34-40]. The effects of PKA and PKC activators on spinal transmission and excitability were blocked by inhibitors of ERK signaling, suggesting that PKA and PKC are upstream activators of ERK in the spinal cord [39,40].

Pain-related functions and interactions of protein kinases, including PKA, PKC, and ERK, in the amygdala are largely unknown. A recent biochemical and behavioral study showed ERK activation in the CeLC in the formalin pain model and antinociceptive effects of inhibiting ERK activation in the CeLC [2]. The present study used a multidisciplinary approach at the cellular and system levels to determine the effects of selective inhibitors of PKA, PKC, and ERK in the amygdala on pain-related synaptic plasticity and behavior. We focused on these protein kinases because they are important for spinal central sensitization and can phosphorylate the NMDA receptor [41-43], which is a critical mechanism of arthritis pain-related plasticity in the amygdala [17].

## Methods

All experimental procedures were approved by the Institutional Animal Use and Care Committee (IACUC) at the University of Texas Medical Branch and conform to guidelines of the International Association for the Study of Pain (IASP) and of the National Institutes of Health (NIH). Electrophysiological and behavioral data were obtained from normal rats (n = 34) and from rats with an acute monoarthritis (n = 60; see below). Adult male Sprague-Dawley rats (120–250 g) were used for all experiments, 94 animals in total. Rats were individually housed in standard plastic boxes (40 × 20 cm) in a temperature-controlled room and maintained on a 12 h day and night cycle. Standard laboratory chow and tap water was continuously available. On the day of the experiment, rats were transferred from the animal facility and allowed to acclimate to the laboratory for at least 1 h.

### Arthritis pain model

In some animals (n = 60) a localized mono-arthritis was induced in the left knee. For arthritis induction, animals were briefly (for 20 min) anesthetized either with the short-acting barbiturate sodium methohexital (Brevital^®^, 50 mg/kg, i.p.) or with 5% isoflurane (1-chloro-2,2,2-trifluoroethyl difluoromethyl ether; Hospira Inc. Lake Forest, IL, USA) using an Ohio Isoflurane Vaporizer (100F model). Since no differences in electrophysiological and behavioral changes were found, data were pooled. A kaolin suspension (4%, 80–100 μl) was injected into the joint cavity through the patellar ligament with a syringe (1 ml, 25 G5/8). After repetitive flexions and extensions of the knee for 15 min, a carrageenan solution (2%, 80–100 μl) was injected into the knee joint cavity and the leg was flexed and extended for another 5 min. This treatment paradigm reliably leads to inflammation and swelling of the knee within 1–3 h, reaches a maximum plateau at 5–6 h, and persists for days [44]. The monoarthritis is strictly confined to the knee; it does not spread and become systemic; and it is a use-dependent pain model, i.e., signs of "spontaneous" pain are typically not observed in the absence of external stimulation or movement [44]. Animals recovered quickly and were closely monitored for any signs of distress, using a "Quantitative Assessment for Pain and Distress Chart" provided by our IACUC. Parameters included overall appearance, breathing patterns, grooming behavior, locomotion around the cage, water and food consumption, spontaneous vocalizations, and interactions with the investigator. Electrophysiological and behavioral measurements of arthritis pain-related changes were made 6 h after arthritis induction (plateau phase, see above). During the development of arthritis the animals were without the benefit of anesthesia or analgesics. The addition of any analgesics or continuous anesthesia would preclude the measurement of pain-related behavior and affect neuronal activity in the brain slice by chemical contamination to such extent that it would invalidate the data and confound the results and interpretation.

### Electrophysiology: patch clamp recording

#### Amygdala slice preparation

Brain slices containing the central nucleus of the amygdala (CeA) were obtained from 51 rats. Rats were decapitated without the use of anesthesia to avoid chemical contamination of the tissue. The brain was quickly dissected out and blocked in cold (4°C) artificial cerebrospinal fluid (ACSF). ACSF contained (in mM): NaCl 117, KCl 4.7, NaH_2_PO_4 _1.2, CaCl_2 _2.5, MgCl_2 _1.2, NaHCO_3 _25, and glucose 11. ACSF was oxygenated and equilibrated to pH 7.4 with a mixture of 95% O_2_/5% CO_2_. Coronal brain slices (500 μm) were prepared using a Vibroslice (Camden Instruments, London, UK). After incubation in ACSF at room temperature (21°C) for at least 1 h, a single brain slice was transferred to the recording chamber and submerged in ACSF (31 ± 1°C), which perfused the slice at a rate of ~2 ml/min. Only 1–2 brain slices per animal were used, and only 1 neuron was recorded in each slice. Unless otherwise stated, numbers in the manuscript refer to the number of neurons tested for each parameter.

#### Whole-cell patch-clamp recording

Recordings were made in the right amygdala because our previous electrophysiological in vivo and in vitro studies showed pain-related plasticity in the right amygdala [10,11,15-17,45] and our behavioral data indicated that the right amygdala is coupled to pain facilitation in the arthritis pain model [10,22,45]. This is consistent with a strong contralateral projection of the spino-parabrachio-amygdaloid pain pathway [1,46] (arthritis was induced in the left knee).

Whole-cell recordings using the "blind" patch technique were obtained from neurons in the latero-capsular division of the CeA (CeLC) as described before [10,16-19]. The different nuclei of the amygdala and the CeA subdivisions are easily discerned under the microscope. Patch electrodes (4–6 MΩ tip resistance) were made from borosilicate glass capillaries (1.5 mm and 1.12 mm, outer and inner diameter, respectively; Drummond, Broomall, PA), using a Flaming-Brown micropipette puller (P-80/PC, Sutter Instrument Co., Novato, CA). The internal solution of the recording electrodes contained (in mM): 122 K-gluconate, 5 NaCl, 0.3 CaCl_2_, 2 MgCl_2_, 1 EGTA, 10 HEPES, 5 Na_2_-ATP, 0.4 Na_3_-GTP; pH was adjusted to 7.2–7.3 with KOH and the osmolarity to 280 mOsm/kg with sucrose. After tight (>2 GΩ) seals were formed and the whole-cell configuration was obtained, neurons were included in the sample if the resting membrane potential was more negative than -50 mV and action potentials overshooting 0 mV were evoked by direct depolarizing current injections.

Voltage and current signals were low-pass filtered at 1 kHz with a dual 4-pole Bessel filter (Warner Instrument Corp., Hamden, CT), digitized at 5 kHz (Digidata 1322A interface, Axon Instr., Molecular Devices, Sunnyvale, CA), and stored on a computer (Dell Pentium 4). Data were also continuously recorded on an ink chart recorder (Gould 3400, Gould Instr., Valley View, OH). Current- and voltage-clamp (d-SEVC) recordings were made using an Axoclamp-2B amplifier (Axon Instr.) with a switching frequency of 5–6 kHz (30% duty cycle), gain of 3–8 nA/mV, and time constant of 20 ms. Phase shift and anti-alias filter were optimized. The headstage voltage was monitored continuously on a digital oscilloscope (Gould 400, Gould Instr.) to ensure precise performance of the amplifier. If series resistance (monitored with pCLAMP9 software, Axon Instr.) changed more than 10%, the neuron was discarded. Voltage- and current data were analyzed with pCLAMP9 software (Axon Instruments). Neurons were voltage-clamped at -60 mV except for the analysis of NMDA receptor-mediated synaptic transmission (recorded at +20 mV).

#### Synaptic stimulation

**Figure 1:**
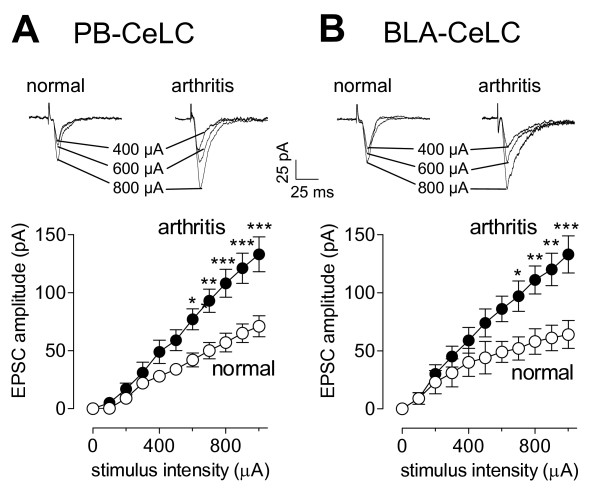
**Synaptic plasticity in CeLC neurons in the arthritis pain model**. Input-output functions of CeLC neurons were measured in slices from arthritic animals (6 h postinduction) and in slices from normal animals. Monosynaptic excitatory postsynaptic currents (EPSCs) were evoked in CeLC neurons by electrical stimulation of the PB-CeLC (**A**) and BLA-CeLC (**B**) synapses with increasing intensities. Input-output curves were generated by plotting peak EPSC amplitude (pA) as a function of afferent fiber volley stimulus intensity (μA). Input-output functions of neurons from arthritic animals (n = 19) were significantly different from those of control neurons (n = 16) at the PB-CeLC (P < 0.0001, F _1,363 _= 66.65) and BLA-CeLC (P < 0.0001, F _1,363 _= 43.30, two-way ANOVA) synapses. Individual traces (mean of 8–10 trials) show monosynaptic EPSCs recorded in one CeLC neuron from a normal rat and another CeLC neuron from an arthritic rat. Whole-cell voltage-clamp recordings were made at -60 mV. * P < 0.05, ** P < 0.01, *** P < 0.001 (Bonferroni post-tests).

Using two concentric bipolar stimulating electrodes (SNE-100, Kopf Instr.; 22 kΩ), monosynaptic excitatory postsynaptic currents (EPSCs) were evoked in CeLC neurons by electrical stimulation (using a Grass S88 stimulator) of two distinct lines of input [1,3,8,21]: the PB-CeLC synapse, which contains afferents from the lateral parabrachial area and provides presumed nociceptive input from the spino-parabrachio-amygdaloid pain pathway, and the BLA-CeLC synapse, which transmits highly integrated polymodal information from thalamic and cortical areas and is part of the fear/anxiety circuitry. For stimulation of the PB-CeLC synapse, the electrode was positioned under microscopic control on the afferent fiber tract from the lateral PB, which runs dorsomedial to the CeA and ventral to but outside of the caudate-putamen. In the vicinity of this tract, no other afferents to the CeA have been described [47-49]. Electrical stimuli (150 μs square-wave pulses) were delivered at low frequencies (<0.25 Hz). Input-output functions were obtained by increasing the stimulus intensity in 100 μA steps (see Figure 1). For evaluation of a drug effect on synaptically evoked responses, the stimulus intensity was adjusted to 75–80% of the intensity required for orthodromic spike generation [10,16,17].

#### Drugs

The following membrane-permeable selective protein kinase inhibitors were used: (9R,10S,12S)-2,3,9,10,11,12-hexahydro-10-hydroxy-9-methyl-1-oxo-9,12-epoxy-1H-diindolo [1,2,3-fg:3',2',1'-kl]pyrrolo [3,4-i][1,6]benzodiazocine-10-carboxylic acid, hexyl ester (KT5720, a potent and selective PKA inhibitor[17,50]); (R)-adenosine, cyclic 3',5'-(hydrogenphosphorothioate) triethylammonium (cAMPS-Rp, a competitive cAMP antagonist[51]); 1,4-diamino-2,3-dicyano-1,4-bis(2-aminophenylthio)butadiene (U0126, a selective ERK inhibitor[52]); 1,4-diamino-2,3-dicyano-1,4-bis(methylthio)butadiene (U0124, inactive analogue of U0126 [52]); and 2-[1-(3-dimethylaminopropyl)indol-3-yl]-3-(indol-3-yl) maleimide (GF109203x, a potent and selective PKC inhibitor[53]). For the analysis of NMDA receptor-mediated transmission we used DL-2-amino-5-phosphonopentanoic acid (AP5, NMDA receptor antagonist), 2,3-dioxo-6-nitro-1,2,3,4-tetrahydrobenzo [f]quinoxaline-7-sulfonamide disodium salt (NBQX, non-NMDA receptor antagonist), and [R-(R*,S*)]-6-(5,6,7,8-tetrahydro-6-methyl-1,3-dioxolo [4,5-g]isoquinolin-5-yl)furo [3,4-e]-1,3-benzodioxol-8(6H)-one (bicuculline, GABA_A_ receptor antagonist). All drugs were purchased from Tocris Bioscience, Ellisville, MO, USA. Drugs were dissolved in ACSF on the day of the experiment and applied to the brain slice by gravity-driven superfusion in the ACSF (~2 ml/min). Solution flow into the recording chamber (1 ml volume) was controlled with a three-way stopcock. Drugs were applied for at least 15 min to establish equilibrium in the tissue. Based on our previous studies and initial observations showing that drug effects reached a plateau after 10 min, the 12–15 min time point was selected for the analysis of drug effects on synaptic transmission. In some experiments KT5720 was included in the internal pipette solution for direct application into the cell.

### Behavior: vocalizations and hindlimb withdrawal reflexes

For behavioral tests 43 rats were used.

#### Experimental protocol

A guide cannula for ACSF and drug application by microdialysis was stereotaxically implanted in the CeLC (or striatum as placement control). The next day, vocalizations and spinal withdrawal reflexes were measured in 2 groups of animals. Rats in the "normal" group were tested before (ACSF vehicle control) and during drug administration (15 min). The "arthritis" group was tested before arthritis induction (baseline), 6 h postinduction of arthritis (ACSF vehicle predrug control), during drug administration (15 min), and after drug administration (30 min washout with ACSF). ACSF served as a vehicle control because drugs were dissolved in ACSF. Arthritis was induced as described under "Arthritis pain model". At the end of the experiment, the animal was sacrificed by decapitation under anesthesia with pentobarbital (Nembutal, 50 mg/kg, i.p.).

#### Microdialysis for drug application

Drugs were administered into the right CeLC contralateral to the arthritis (see 2.2.2). As described in detail before [9,10,22], rats were anaesthetized with pentobarbital sodium (50 mg/kg, i.p.) and a small unilateral craniotomy was performed at the sutura fronto-parietalis level. Using a stereotaxic apparatus (David Kopf Instr., Tujunga, CA), a guide cannula was implanted on the dorsal margin of the CeLC using the following coordinates [54]: 2.0 ± 0.1 mm caudal to bregma, 4.0 mm lateral to midline, depth 7.0 mm. In some experiments a guide cannula was implanted into the striatum as a placement control, using the following stereotaxic coordinates 2.0 ± 0.1 mm caudal to bregma; 4.5 mm lateral to midline; depth of tip 5.0 mm. The cannula was fixed to the skull with dental acrylic (PlasticsOne, Roanoke, VA). Antibiotic ointment was applied to the exposed tissue to prevent infection. On the day of the experiment a microdialysis probe (CMA/Microdialysis 11; membrane diameter: 250 μm, membrane length: 1 mm) was inserted into the CeLC through the guide cannula so that the probe protruded by 1 mm. Using PE-50 tubing, the probe was connected to a Harvard infusion pump and perfused with ACSF (2 μl/min) containing (in mM): NaCl 125.0, KCl 2.6, NaH_2_PO_4 _2.5, CaCl_2 _1.3, MgCl_2 _0.9, NaHCO_3 _21.0, and glucose 3.5; oxygenated and equilibrated to pH = 7.4. Before each drug application, ACSF was pumped through the fiber for at least 1 h to establish equilibrium in the tissue.

#### Drugs

Protein kinase inhibitors (same as in Electrophysiology) were dissolved in ACSF on the day of the experiment at a concentration 100 times that predicted to be needed based on published biochemical data [50,51,53], our previous microdialysis study[33], and our in vitro data [[10]; and this study, [17]] because of the concentration gradient across the dialysis membrane and diffusion in the tissue [10-14]. The numbers given in this article refer to the drug concentrations in the microdialysis fiber. ACSF administered alone served as a vehicle control. Behavior was measured at 15 min during continued drug administration and again at 30 min of washout with ACSF.

#### Audible and ultrasonic vocalizations

Vocalizations were recorded and analyzed as described in detail previously [22]. The experimental setup (US Patent 7,213,538) included a custom designed recording chamber, a condenser microphone (audible range: 20 Hz-16 kHz) connected to a preamplifier, an ultrasound detector (25 ± 4 kHz), filter and amplifier (UltraVox 4-channel system, Noldus Information Technology, Leesburg, VA). Data acquisition software (UltraVox 2.0; Noldus Information Technology) automatically monitored the occurrence of vocalizations within user-defined frequencies and recorded the number and duration of digitized events (audible and ultrasonic vocalizations). Audible and ultrasonic vocalizations were recorded simultaneously with the two microphones connected to separate channels of the amplifier. The computerized recording system was set to suppress non-relevant audible sounds (background noise) and to ignore ultrasounds outside the defined frequency range.

Animals were placed in the recording chamber for acclimation 1 h before the vocalization measurements. The recording chamber ensured the stable positioning of the animal at a fixed distance from the sound detectors and allowed the reproducible stimulation of the knee joint through openings for the hind limbs. Brief (15 s) innocuous (100 g/30 mm^2^) and noxious (2000 g/30 mm^2^) mechanical stimuli were applied to the knee, using a calibrated forceps equipped with a force transducer, the output of which was displayed on an LCD screen [10,55]. The chamber also had an opening for drug administration through the microdialysis probe inserted into the implanted guide cannula. The total duration of vocalizations (arithmetic sum of the duration of individual events) was recorded for 1 min, starting with the onset of the mechanical stimulus. Audible and ultrasonic vocalizations reflect supraspinally organized nocifensive and affective responses to aversive stimuli [44,56].

#### Hindlimb withdrawal reflex

Thresholds of spinal withdrawal reflexes evoked by mechanical stimulation of the knee joint were measured subsequently to the vocalization measurements as described in detail before [44,55]. Mechanical stimuli of continuously increasing intensity were applied to the knee joint, using a calibrated forceps with a force transducer as in the vocalization experiments. Withdrawal threshold was defined as the minimum stimulus intensity that evoked a withdrawal reflex.

### Histology

At the end of each behavioral experiment, the position of the microdialysis probe in the CeLC or striatum (placement control) was confirmed histologically. The brain was removed and submerged in 10% formalin. Tissues were stored in 20% sucrose before they were frozen sectioned at 50 μm. Sections were stained with Neutral Red, mounted on gel-coated slides and cover-slipped. Lesion sites were plotted on standard diagrams.

### Data analysis and statistics

All averaged values are given as the mean ± SEM. Statistical significance was accepted at the level P < 0.05. GraphPad Prism 3.0 software (GraphPad Software Inc., San Diego, CA) was used for all statistical analyses.

#### Electrophysiology

Input-output functions were compared using repeated-measures two-way analysis of variance (ANOVA) followed by Bonferroni post-tests. The paired t-test was used to compare evoked EPSC amplitudes before and after a single drug application in the same neuron. Time-course data of the effects of a single drug were compared to predrug values in the same neuron using repeated-measures ANOVA followed by Dunnett's Multiple Comparison Test. Repeated-measures ANOVA followed by Bonferroni post-tests was used to compare the effects of more than one drug in the same neuron to predrug control values and to each other.

#### Behavior

The duration of audible and ultrasonic vocalizations was calculated as the arithmetic sum (total amount) of the durations of individual vocalization events in a 1 min recording period. Vocalizations and withdrawal thresholds of the same animal before and after arthritis and before and during drug application in arthritis were compared using repeated-measures ANOVA followed by Bonferroni post-tests ("arthritis" group, see Experimental protocol). The paired t-test was used to compare behavior before and during drug administration in normal animals ("normal" group).

## Results

### Enhanced synaptic transmission in CeLC neurons in the arthritis pain model

Whole-cell patch-clamp recordings were made of neurons in the latero-capsular division of the central nucleus of the amygdala (CeLC) in brain slices from normal rats (n = 26 neurons) and from arthritic rats (6 h postinduction; n = 35 neurons) in 51 animals. Recordings were made in the right amygdala because of the strong contralateral projection of the spino-parabrachio-amygdaloid pain pathway [1,46] (arthritis was induced in the left knee). The right amygdala develops pain-related plasticity [10,11,15-17,45] and is coupled to pain facilitation [10,22,45] in the arthritis pain model.

Only 1 or 2 brain slices per animal were used and 1 neuron was recorded in each slice. Like most CeLC neurons [1,3,21], all CeLC neurons in this study responded to electrical stimulation of the PB-CeLC synapse and the BLA-CeLC synapse (see "Synaptic stimulation" in Methods). Based on their action potential firing properties these neurons were non-accommodating repetitive- and regular-spiking, which is the prevalent type of neurons in this division of the amygdala [16,57-59].

In agreement with our previous studies [10,16,17,19] enhanced synaptic transmission was recorded in the CeLC in brain slices from arthritic rats compared to control CeLC neurons from normal rats (Figure 1). Analysis of input-output functions showed increased synaptic strength measured as increased peak amplitudes of monosynaptic excitatory postsynaptic currents (EPSCs) evoked at the PB-CeLC synapse (Figure 1A; P < 0.0001, F _1,363 _= 66.65) and the BLA-CeLC synapse (Figure 1B; P < 0.0001, F _1,363 _= 43.30, two-way ANOVA) in the arthritis pain model (n = 19 neurons) compared to normal transmission (n = 16 neurons). Enhanced synaptic transmission preserved in the slice preparation indicates "synaptic plasticity" because it is maintained independently of peripheral or spinal mechanisms [1].

### Inhibition of PKA activation decreases pain-related synaptic plasticity

**Figure 2:**
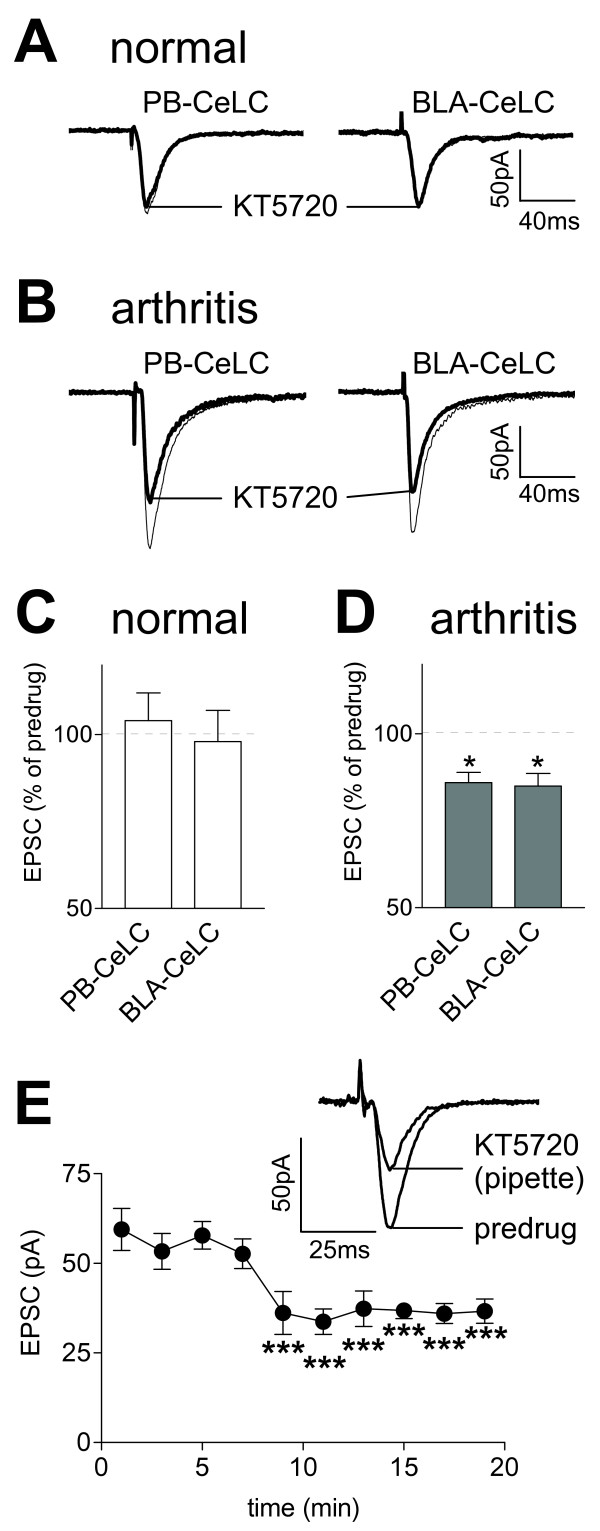
**A PKA inhibitor (KT5720) inhibits synaptic plasticity but not normal synaptic transmission**. Monosynaptic EPSCs were evoked at the PB-CeLC and BLA-CeLC synapses in slices from normal (**A, C**) and arthritic (6 h postinduction; **B, D, E**) rats. KT5720 (1 μM, 15 min) inhibited synaptic transmission in neurons from arthritic rats 6 h postinduction (**D**, n = 7; P < 0.05, paired t-test) but not in control neurons from normal rats (**C**, n = 7). (**A, B**) Original recordings of EPSCs (average of 8–10 EPSCs) evoked at the two synapses. (**C, D**) Averaged EPSC amplitudes (mean ± SE) in the presence of KT5720 normalized to predrug (ACSF) control values (set to 100%). (**E**) Time course of the inhibitory effect of direct intracellular application of KT5720 (1 μM) through the patch pipette. Each symbol shows averaged EPSC amplitudes (mean ± SE; n = 4) at different times after whole-cell configuration was obtained (t = 0). The inhibitory effect was significant (P < 0.001, compared to the first EPSC after patch formation; Dunnett's Multiple Comparison Test). Insets show EPSCs (average of 8–10 trials) evoked at the PB-CeLC synapse at 1 min (predrug) and at 15 min (KT5720) after patch formation. Whole-cell voltage-clamp recordings were made at -60 mV. * P < 0.05, *** P < 0.001.

A selective membrane-permeable PKA inhibitor (KT5720) that binds to the catalytic subunits of the cAMP dependent PKA was used [17,50,60]. KT5720 (1 μM, 15 min) decreased the amplitudes of monosynaptic EPSCs evoked at the PB-CeLC and BLA-CeLC synapses in neurons recorded in slices from arthritic rats (n = 7; Figure 2B,D) but not in control neurons from normal rats (n = 7; Figure 2A,C). The inhibitory effect of KT5720 was significant compared to predrug (ACSF) control values obtained in the same neurons (P < 0.05, paired t-test, Figure 2D). To confirm that the effect of KT5720 was due to a direct action inside the CeLC neurons, KT5720 was applied into the cell through the patch pipette filled with internal solution containing KT5720 (1 μM; Figure 2E). Monosynaptic EPSCs evoked at the PB-CeLC synapse were measured immediately after whole-cell patch configuration was obtained (n = 4 neurons). EPSC amplitude decreased 9 min after the patch formation when the PKA inhibitor had entered the cell. The inhibitory effect of intracellularly applied KT5720 was significant compared to the control value obtained immediately after the patch formation (P < 0.001, repeated measures ANOVA followed by Dunnett's Multiple Comparison Test).

**Figure 3:**
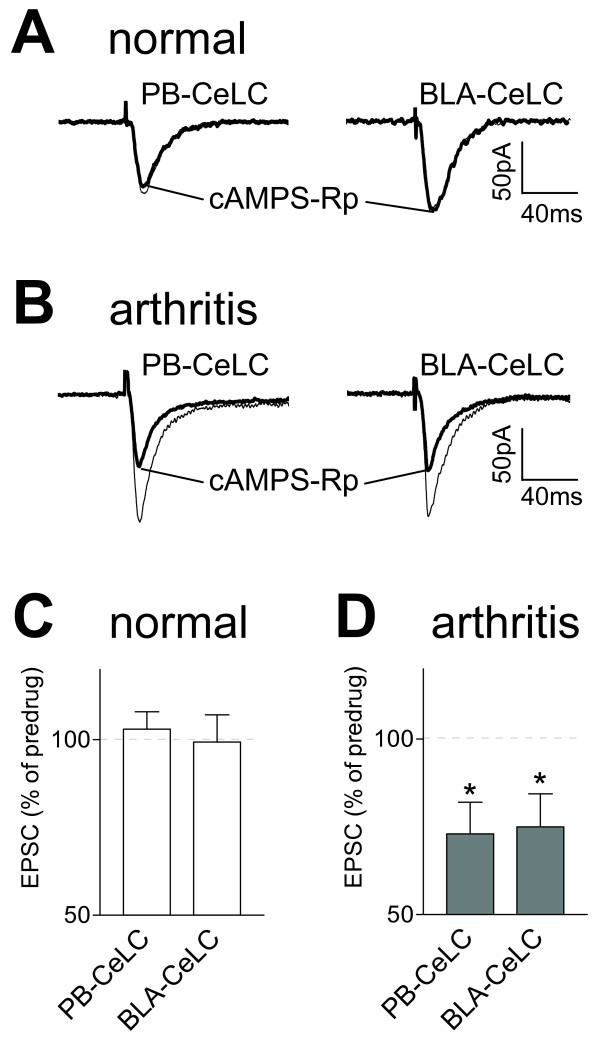
**A cAMP antagonist (cAMPS-Rp) inhibits synaptic plasticity but not normal synaptic transmission**. Monosynaptic EPSCs were evoked at the PB-CeLC and BLA-CeLC synapses in slices from normal (**A, C**) and arthritic (6 h postinduction; **B, D**) rats. cAMPS-Rp (10 μM, 15 min) inhibited synaptic transmission in neurons from arthritic rats 6 h postinduction (**D**, n = 6; P < 0.05, paired t-test) but not in control neurons from normal rats (**C**, n = 4). (**A, B**) Original recordings of EPSCs (average of 8–10 EPSCs) evoked at the two synapses. (**C, D**) Averaged EPSC amplitudes (mean ± SE) in the presence of cAMPS-Rp normalized to predrug control values (set to 100%). Whole-cell voltage-clamp recordings were made at -60 mV. * P < 0.05.

A membrane-permeable competitive cAMP antagonist (cAMPS-Rp) that blocks PKA activation by binding to the regulatory subunits without dissociating the kinase holoenzyme [51] also inhibited synaptic plasticity (Figure 3B,D) but had no effect on normal synaptic transmission (Figure 3A,C). cAMPS-Rp (10 μM, 15 min) decreased the monosynaptic EPSCs evoked at the PB-CeLC and BLA-CeLC synapses in slices from arthritic rats (n = 6 neurons) but not in control neurons from normal animals (n = 4). The inhibitory effect of cAMPS-Rp was significant compared to predrug (ACSF) control values obtained in the same neurons (P < 0.05, paired t-test, Figure 3D).

### Inhibition of ERK activation decreases pain-related synaptic plasticity

**Figure 4:**
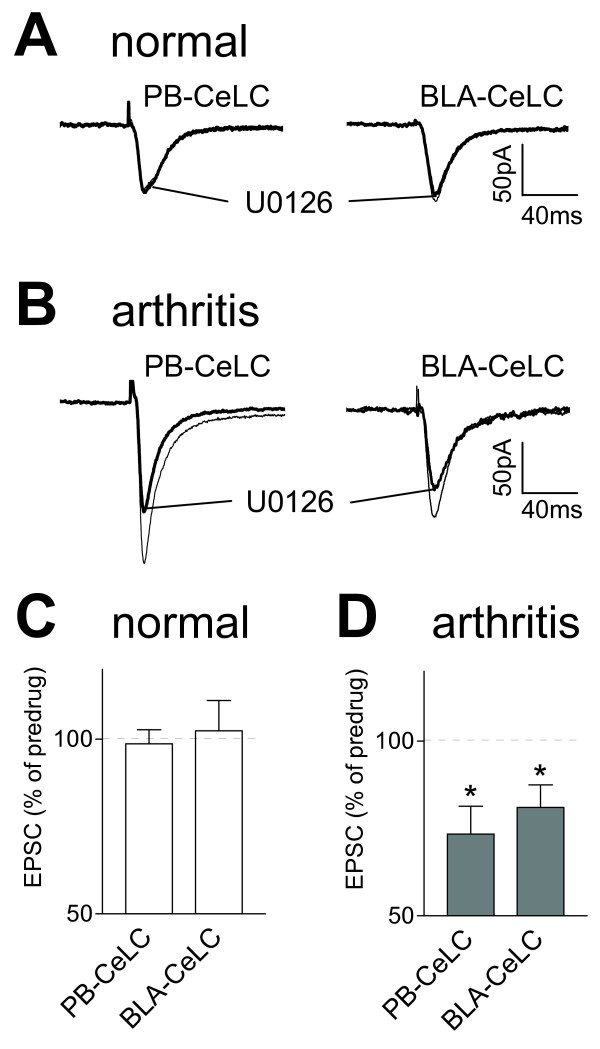
**An ERK inhibitor (U0126) inhibits synaptic plasticity but not normal synaptic transmission**. Monosynaptic EPSCs were evoked at the PB-CeLC and BLA-CeLC synapses in slices from normal (**A, C**) and arthritic (6 h postinduction; **B, D**) rats. U0126 (1 μM, 15 min) inhibited synaptic transmission in neurons from arthritic rats 6 h postinduction (**D**, n = 6; P < 0.05, paired t-test) but not in control neurons (**C**, n = 6). (**A, B**) Original recordings of EPSCs (average of 8–10 trials) evoked at the two synapses. (**C, D**) Averaged EPSC amplitudes (mean ± SE) in the presence of U0126 normalized to predrug control values (set to 100%). Whole-cell voltage-clamp recordings were made at -60 mV. * P < 0.05.

A recent behavioral study showed antinociceptive effects of an ERK inhibitor administered into the CeLC [2]. However, the contribution of ERK to synaptic transmission and plasticity in the CeLC is unknown. We used a membrane-permeable selective inhibitor of ERK activation (U0126) and its inactive structural analogue (U0124, see below) [52]. U0126 (1 μM, 15 min) inhibited synaptic plasticity in neurons from arthritic rats (n = 6 neurons; Figure 4B,) but had no effect on basal synaptic transmission in neurons from normal rats (n = 6; Figure 4A,C). The inhibition of synaptic plasticity by U0126 was significant compared to predrug (ACSF) control values obtained in the same neurons (P < 0.05, paired t-test; Figure 4D).

### PKA and ERK inhibitors have additive effects on NMDA receptor-mediated synaptic transmission

**Figure 5:**
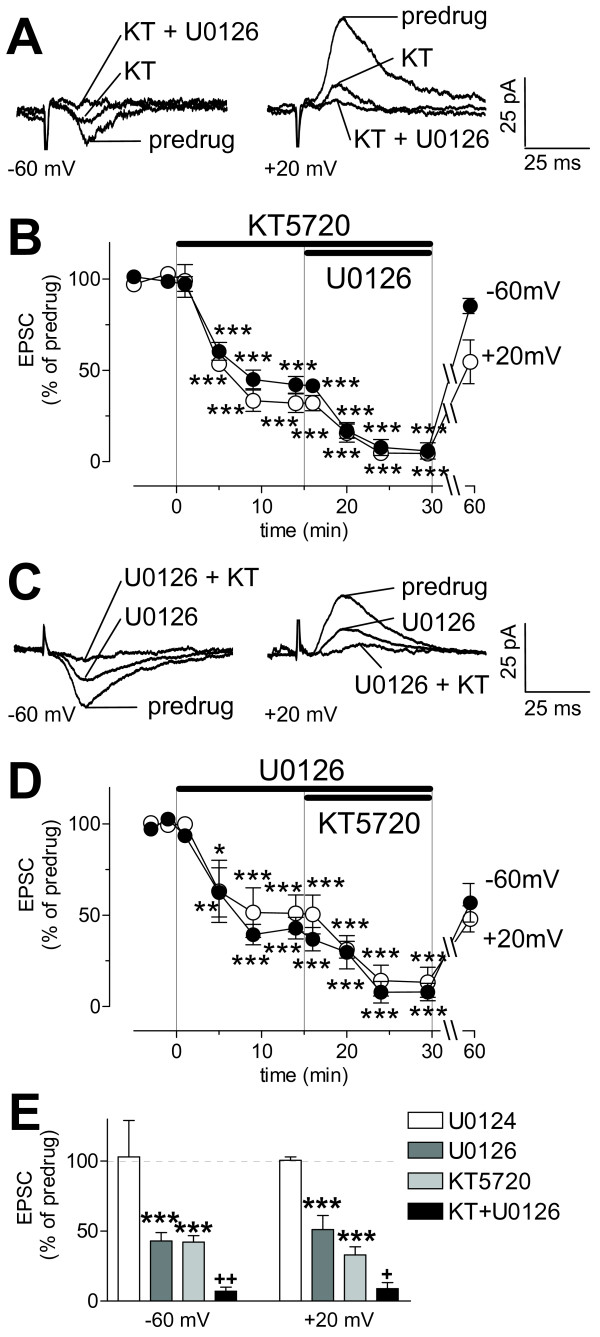
**Additive effect of PKA and ERK inhibitors on NMDA receptor-mediated synaptic plasticity**. **(A, B) **KT5720 (KT, 1 μM, 15 min; n = 3) inhibited the pharmacologically (with NBQX, 20 μM; bicuculline, 30 μM) isolated NMDA receptor-mediated synaptic component in slices from arthritic rats (P < 0.001, repeated-measures ANOVA with Bonferroni post-tests). Co-application of KT5720 and U0126 (1 μM, n = 3) further decreased the synaptic response (P < 0.001, Bonferroni post-tests). **(C, D) **U0126 (1 μM, 15 min) applied alone (n = 3) or together with KT5720 (n = 3) inhibited the NMDA component in the arthritis pain model (P < 0.05–0.001, Bonferroni post-tests). **(E) **Coapplication of KT5720 and U0126 (n = 6) had a significantly greater effect than each compound alone (P < 0.05–0.01, one-way ANOVA with Bonferroni post-tests). A negative structural analogue of U0126 (U0124, 1 μM, n = 3) had no effect. Bar histograms show averaged EPSC amplitudes (mean ± SE) normalized to predrug control values (set to 100%). **(A, C) **Monosynaptic EPSCs (average of 8–10 traces) recorded at -60 mV and +20 mV. **(B, D) **Time course of drug effects at -60 mV and +20 mV. Each symbol shows averaged EPSC amplitudes (mean ± SE) normalized to predrug control (set to 100%). * P < 0.05, ** P < 0.01, *** P < 0.001 (compared to predrug values); + P < 0.05, ++ P < 0.01 (compared to each drug alone).

NMDA receptors mediate synaptic plasticity in the CeLC in the arthritis pain model but do not contribute to basal synaptic transmission under normal conditions [17]. PKA and ERK inhibitors selectively affect synaptic plasticity but not normal transmission (Figures 2, 3, 4) and can phosphorylate NMDA receptors [41-43]. Therefore, we hypothesized that NMDA receptors were the target of these protein kinases. KT5720 (1 μM, 15 min) inhibited the pharmacologically (with NBQX, 20 μM, and bicuculline, 30 μM) isolated NMDA receptor-mediated synaptic component in the arthritis pain model (Figure 5A, individual example; 5B, time course and summary, n = 3 neurons). The inhibitory effect was significant (P < 0.001, compared to predrug vehicle control, repeated-measures ANOVA with Bonferroni post-tests). The addition of U0126 further decreased the NMDA receptor-mediated EPSC (P < 0.001, Bonferroni post-tests). The same result was obtained when U0126 was applied first and KT5720 was added subsequently (Figure 5C, individual example; Figure 5D, time course and summary, n = 3 neurons). Inhibition by U0126 and by coapplication of KT5720 and U0126 was significantly different from predrug vehicle control values (P < 0.05–0.001, repeated-measures ANOVA with Bonferroni post-tests). Figure 5E summarizes the results. KT5720 and U0126 applied together (n = 6 neurons) had a significantly greater effect on NMDA receptor-mediated EPSCs than KT5720 or U0126 alone (P < 0.05–0.01, one-way ANOVA with Bonferroni post-tests). The inactive structural analogue of U0126 (U0124, 1 μM, n = 3) had no significant effect. These experiments were done only in slices from arthritic animals because KT5720 and U0126 had no effect on basal synaptic transmission in slices from normal animals (see Figures 2 and 4).

### The effect of PKA activation by forskolin does not depend on ERK

**Figure 6:**
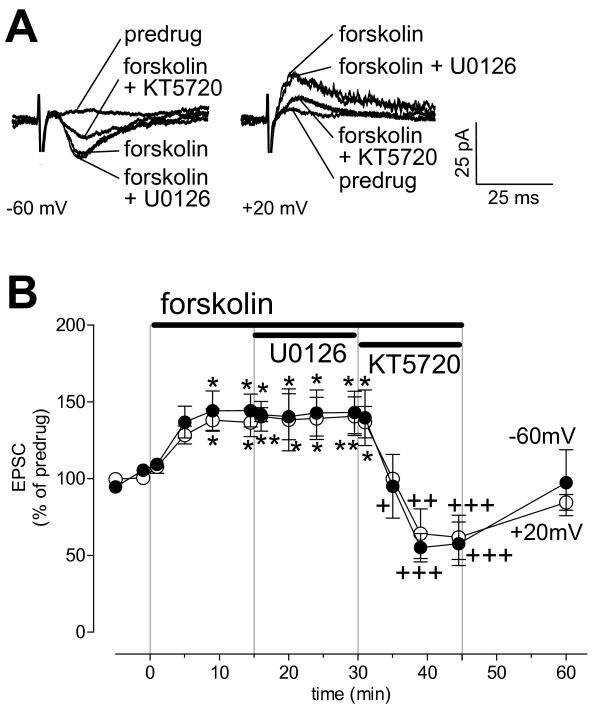
**Synaptic facilitation by forskolin is not impaired by an ERK inhibitor (U0126)**. PKA activation by forskolin (5 μM, 15 min) increased transmission at the PB-CeLC synapse in slices from normal animals and induced an NMDA receptor-mediated component in the presence of NBQX (20 μM) and bicuculline (30 μM). U0126 (1 μM, 15 min) had no effect but KT5720 (1 μM, 1 min) inhibited the forskolin-induced facilitation. **(A) **Monosynaptic EPSCs (average of 8–10 traces) recorded in an individual CeLC neuron held at -60 mV and +20 mV. **(B) **Time course of drug effects at -60 mV and +20 mV (n = 4). Each symbol shows averaged EPSC amplitudes (mean ± SE) normalized to predrug control values (set to 100%). * P < 0.05, ** P < 0.01 (compared to predrug values), + P < 0.05, ++ P < 0.01, +++ P < 0.001 (compared to forskolin without KT5720; repeated-measures ANOVA with Bonferroni post-tests).

The additive effect of PKA and ERK inhibitors suggest that PKA and ERK do not simply act in a serial arrangement in which one inhibitor would occlude the effect of the other. To confirm that PKA activation modulates synaptic transmission independently of ERK activation we measured the effect of U0126 on synaptic facilitation by forskolin-induced PKA activation. These experiments (n = 4 neurons, Figure 6) were done in slices from normal animals to determine if forskolin could mimic the changes observed in the arthritis pain model. Forskolin (5 μM, 15 min) increased synaptic transmission and induced an NMDA receptor-mediated component that is normally weak or absent in control slices but can be observed in slices from arthritic animals [17]. U0126 (1 μM, 15 min) had no effect on the pharmacologically (with NBQX, 20 μM, and bicuculline, 30 μM) isolated NMDA component. KT5720 (1 μM, 1 min) inhibited the synaptic facilitation by forskolin (Figure 6A, individual traces recorded in one CeLC neuron; Figure 6B, time course and summary). These results may suggest that PKA and ERK modulate synaptic transmission through independent signaling mechanisms.

### Inhibition of PKC activation has no effect on pain-related synaptic plasticity

**Figure 7:**
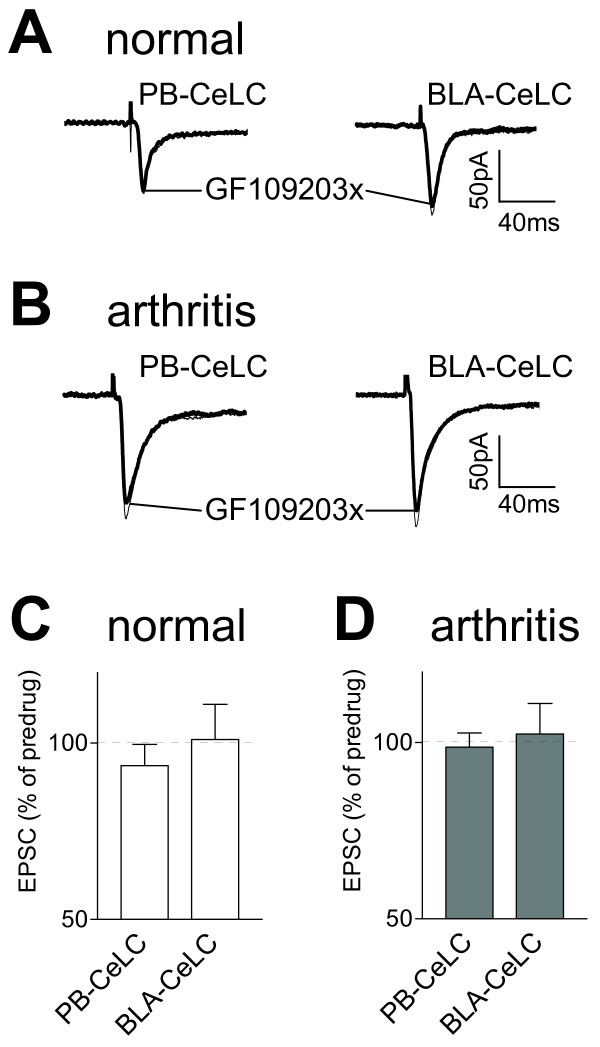
**A PKC inhibitor (GF109203X) has no effect on synaptic transmission**. Monosynaptic EPSCs were evoked at the PB-CeLC and BLA-CeLC synapses in slices from normal (**A, C**) and arthritic (6 h postinduction; **B, D**) rats. GF109203X (1 μM, 15 min) had no significant effect (P > 0.05, paired t-test) in neurons from arthritic rats (n = 6) and in control neurons from normal rats (n = 5). (**A, B**) Original recordings of EPSCs evoked at the two synapses (average of 8–10 EPSCs). (**C, D**) Averaged EPSC amplitudes (mean ± SE) in the presence of GF109203X normalized to predrug control values (set to 100%).

A membrane-permeable selective PKC inhibitor (GF109203X [53]) affected neither normal synaptic transmission (Figure 7A,C) nor synaptic plasticity (Figure 7B,D). GF109203X (1 μM, 15 min) had no significant effect on the monosynaptic EPSCs evoked at the PB-CeLC and BLA-CeLC synapses in slices from normal rats (n = 5 neurons) and in slices from arthritic rats (n = 6; P > 0.05, paired t-test).

### Inhibition of PKA activation decreases pain-related behaviors

**Figure 8:**
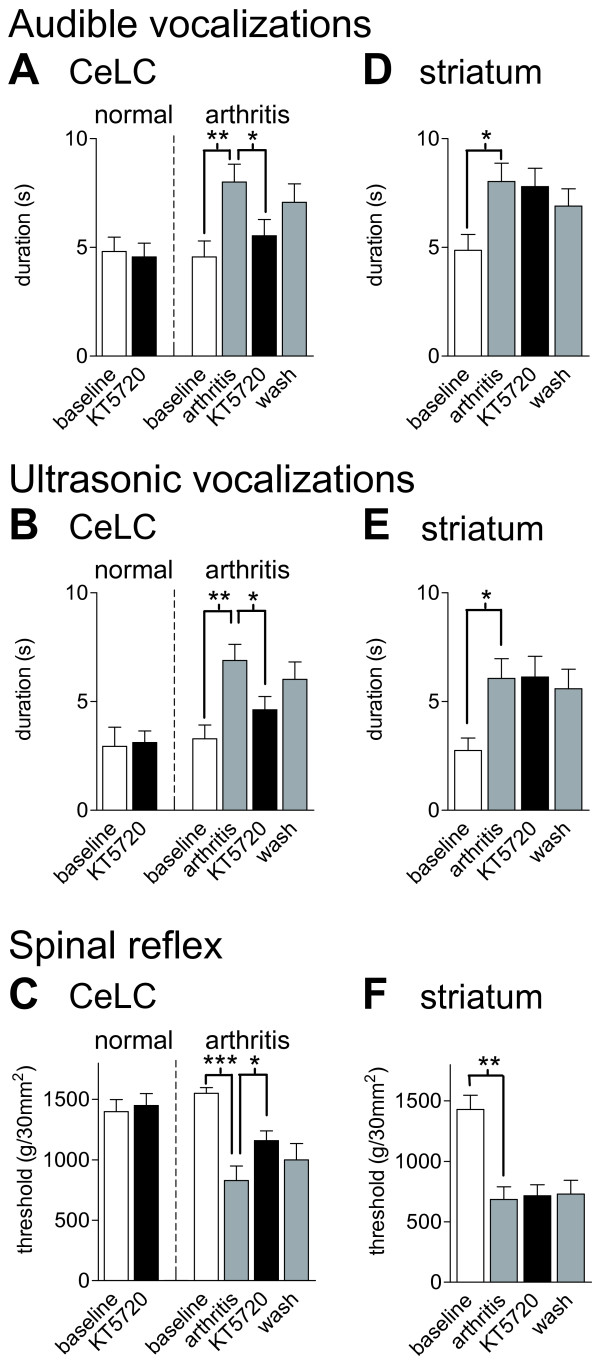
**A PKA inhibitor decreases pain-related behavior in arthritic but not in normal animals**. KT5720 (100 μM, concentration in microdialysis probe, 15 min) administered into the CeLC had no significant effect on audible (**A**) and ultrasonic (**B**) vocalizations and on hindlimb withdrawal reflexes (**C**) in normal animals ("normal"; n = 5). In arthritic animals ("arthritis" in **A-C**; n = 6) KT5720 significantly inhibited vocalizations and increased mechanical thresholds. In the arthritis group, behaviors were measured before (baseline) and 6 h after arthritis induction and during (15 min) and after (30 min washout) drug application. (**D-F**) Administration of KT5720 (100 μM) into the striatum as placement control had no effect on audible and ultrasonic vocalizations and on spinal reflexes of arthritic rats (n = 4). Vocalizations were evoked by brief (15 s) noxious (2000 g/30 mm^2^) stimulation of the knee with a calibrated forceps. Duration of vocalizations was measured as the arithmetic sum of the duration of each individual vocalization event during a 1 min period beginning with the onset of the stimulus (see Methods for details). Bar histograms and error bars show mean ± SE. * P < 0.05, ** P < 0.01, *** P < 0.001 (repeated-measures ANOVA with Bonferroni post-tests).

**Figure 9:**
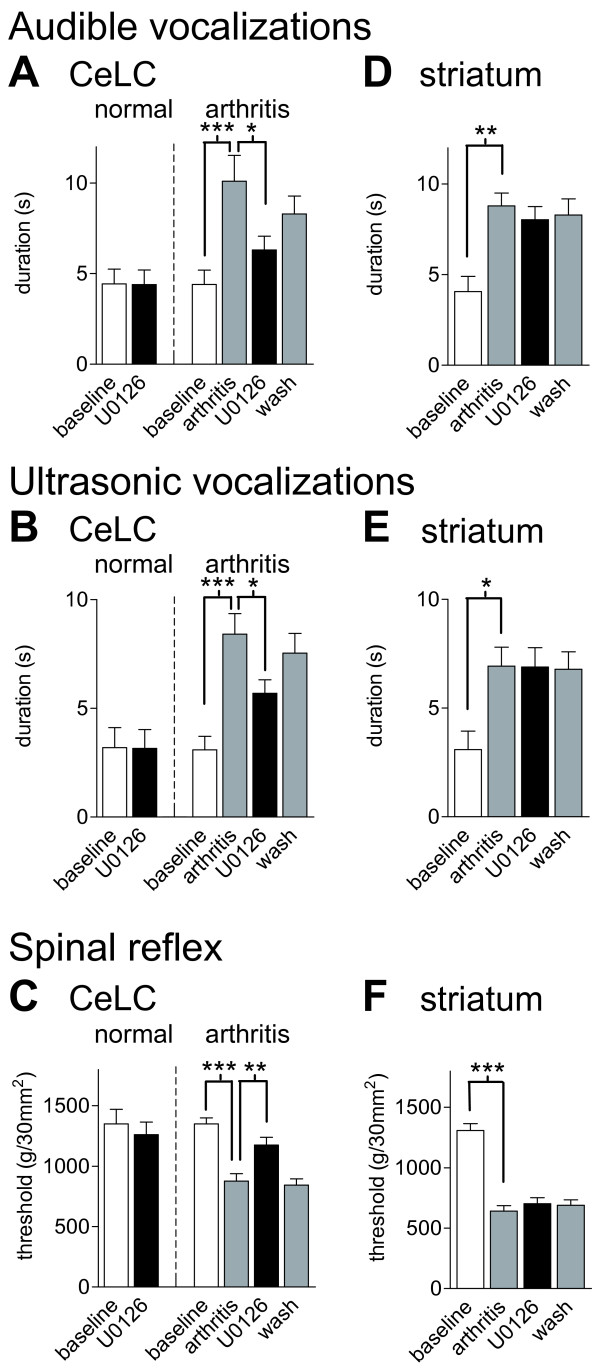
**An ERK inhibitor decreases pain-related behavior in arthritic but not in normal animals**. U0126 (100 μM, concentration in microdialysis probe, 15 min) administered into the CeLC had no significant effect on audible (**A**) and ultrasonic (**B**) vocalizations and on hindlimb withdrawal reflexes (**C**) in normal animals ("normal"; n = 3). In arthritic animals ("arthritis" in **A-C**) U0126 significantly inhibited vocalizations (n = 9) and increased mechanical thresholds (n = 5). In the arthritis group, behaviors were measured before (baseline) and 6 h after arthritis induction and during (15 min) and after (30 min washout) drug application. (**D-F**) Administration of U0126 (100 μM) into the striatum as placement control had no effect (n = 4). Vocalizations and withdrawal reflexes were measured as in Figure 8 (see Methods for details). Bar histograms and error bars represent mean ± SE. * P < 0.05, ** P < 0.01, *** P < 0.001 (repeated-measures ANOVA with Bonferroni post-tests).

**Figure 10:**
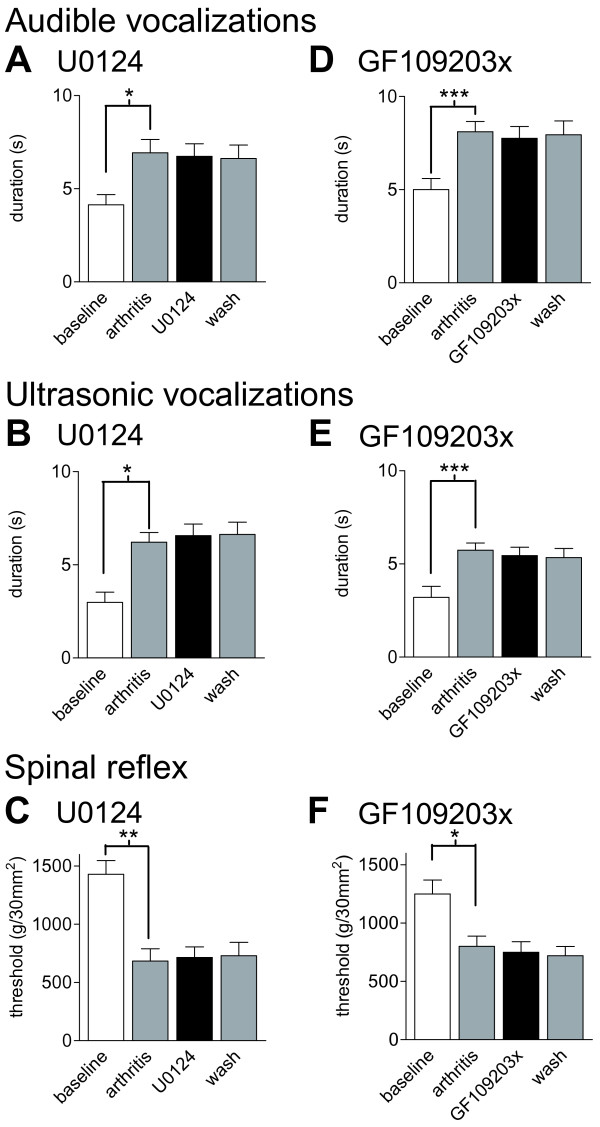
**An inactive structural U0126 analogue (U0124) and a selective PKC inhibitor (GF109203X) have no effect on pain behavior**. (**A-C**) Application of U0124 (100 μM, 15 min) into the CeLC had no effect on the significantly increased audible and ultrasonic vocalizations and on the decreased hindlimb withdrawal thresholds of arthritic rats (n = 3). (**D-F**) GF109203X (100 μM, 15 min) administered into the CeLC had no effect on the significantly increased vocalizations (n = 9) and on decreased withdrawal reflexes (n = 5) of arthritic rats. Vocalizations and withdrawal reflexes were measured as in Figure 8 (see Methods for details). Bar histograms and error bars represent mean ± SE. * P < 0.05, ** P < 0.01, *** P < 0.001 (repeated-measures ANOVA with Bonferroni post-tests).

To validate the significance of the electrophysiological results, we analyzed the effects of protein kinase inhibitors on supraspinally (vocalizations) and spinally (hindlimb withdrawal reflexes) organized pain behaviors in awake animals (Figures 8, 9, 10). Audible (<16 kHz) and ultrasonic (25 ± 4 kHz) vocalizations were evoked by brief (15 s) noxious (2000 g/30 mm^2^) stimulation of the knee with a calibrated forceps. Hindlimb withdrawal reflex thresholds were measured by applying pressure of increasing force to the knee joint with a calibrated forceps. The inhibitors were administered into the CeLC by microdialysis in 2 groups of animals: normal animals without arthritis and arthritic animals (6 h postinduction of arthritis). In the arthritis group, pain behaviors were also measured before arthritis induction to obtain baseline controls. Drugs were administered into the right CeLC contralateral to the arthritis because of the strong contralateral projection of the spino-parabrachio-amygdaloid pain pathway [6] and published data showing that the right CeLC is the site of pain-related plasticity and pain modulation [1,2,8]. All animals had guide cannulas for the microdialysis probes implanted on the day before the behavioral tests (see Methods).

KT5720 (100 μM, concentration in microdialysis probe, 15 min) administered into the CeLC of normal animals (n = 5) had no significant effect on audible (Figure 8A) and ultrasonic (Figure 8B) vocalizations and on hindlimb withdrawal reflexes (Figure 8C). Animals in the arthritis group (n = 6) showed significantly increased vocalizations (P < 0.01) and decreased withdrawal thresholds (P < 0.001, repeated-measures ANOVA with Bonferroni post-tests; Figure 8A–C). KT5720 significantly inhibited audible and ultrasonic vocalizations and significantly increased hindlimb withdrawal thresholds (P < 0.05). The effects were largely reversible after washout (ACSF, 30 min).

Administration of KT5720 (100 μM) into the striatum as placement control had no effect on the significantly increased audible and ultrasonic vocalizations (P < 0.05) and spinal reflexes (P < 0.01, Bonferroni post-tests) of arthritic rats (n = 4; Figure 8D–F). The striatum was chosen as a control site for drug diffusion because it is adjacent (dorsolateral) to the CeLC but does not project to the CeA/CeLC. The distance between the tips of the microdialysis probes in the CeLC and striatum is about 2 mm. We used this placement control successfully in our previous studies [10,22,33]. Placement control experiments were done only in arthritic animals because KT5720 had no effect in the CeLC of normal animals (see Figure 8A–C).

### Inhibition of ERK activation decreases pain-related behaviors

U0126 (100 μM, concentration in microdialysis probe) administered into the CeLC had no significant effect on audible (Figure 9A) and ultrasonic (Figure 9B) vocalizations and on hindlimb withdrawal reflexes (Figure 9C) in normal animals (n = 3). Animals in the arthritis group showed significantly increased vocalizations (n = 9, P < 0.001) and decreased withdrawal thresholds (n = 5, P < 0.001, repeated-measures ANOVA with Bonferroni post-tests; Figure 9A–C). U0126 significantly inhibited audible and ultrasonic vocalizations (P < 0.05) and hindlimb withdrawal reflexes (P < 0.01).

Administration of U0126 (100 μM) into the striatum as placement control had no effect on the significantly increased audible (P < 0.01) and ultrasonic (P < 0.05) vocalizations and spinal reflexes (P < 0.001, Bonferroni post-tests) of arthritic rats (n = 4; Figure 9D–F). Placement control experiments were done only in arthritic animals, because U0126 had no effect in the CeLC of normal animals (Figure 9A–C).

As another control for the selectivity of U0126 the effect of the inactive structural analogue U0124 was tested in arthritic animals (n = 3; Figure 10A–C). Application of U0124 (100 μM, 15 min) into the CeLC had no effect on the significantly increased audible and ultrasonic vocalizations (P < 0.05) and hindlimb withdrawal reflexes (P <0.01, Bonferroni post-tests).

### Inhibition of PKC has no behavioral effect

GF109203X (100 μM, concentration in microdialysis probe) administered into the CeLC had no significant effect on audible (n = 9; Figure 10D) and ultrasonic (n = 9; Figure 10E) vocalizations and on hindlimb withdrawal reflexes (n = 5; Figure 10F) in arthritic animals (6 h postinduction). Animals in the arthritis group showed significantly increased vocalizations (P < 0.001) and withdrawal reflexes (P < 0.05, repeated-measures ANOVA with Bonferroni post-tests). GF109203X was tested only in arthritic animals, because it had no effect on synaptic transmission under normal conditions (see Figure 7A,C).

### Histology

**Figure 11:**
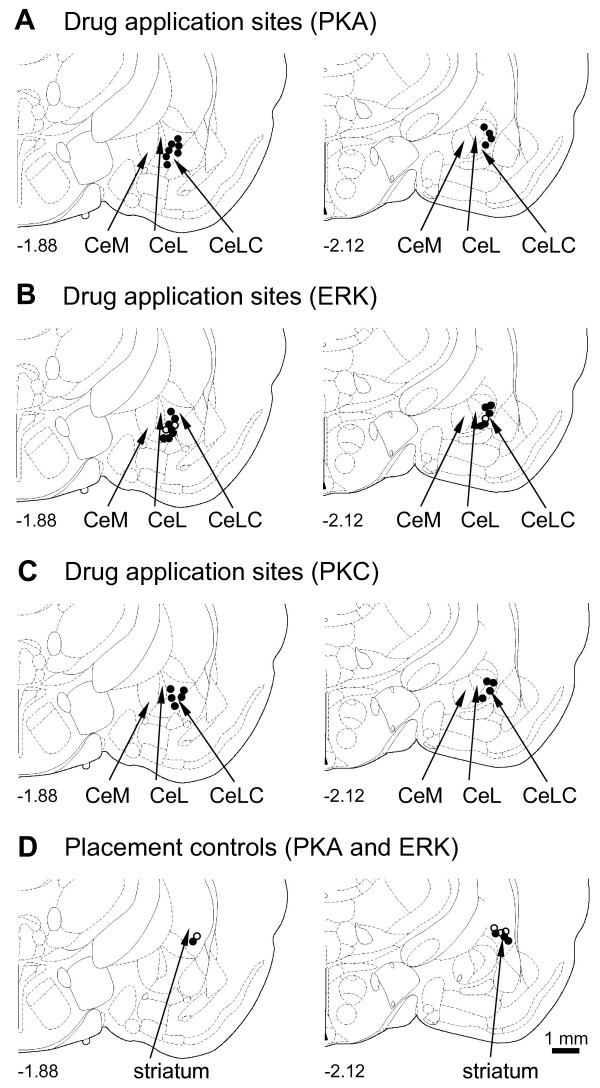
**Histologic verification of drug application sites**. Standard diagrams [adapted from [54]] show coronal sections through the right brain hemisphere at different levels posterior to bregma (-1.88 mm and -2.12 mm). Symbols show the positions of the tips of the microdialysis probe (length of exposed membrane = 1 mm) in the CeLC (**A-C**) and striatum (**D**, placement control) in the behavioral experiments. (**A**) Application sites of KT5720, n = 11. (**B**) U0126, n = 12, filled circles; U0124, n = 3, open circles. (**C**) GF109203X, n = 9. (**D**) KT5720, n = 4, filled circles; U0126, n = 4, open circles. CeM, CeL, CeLC: medial, lateral and laterocapsular divisions of the central nucleus of the amygdala. Calibration bar (1 mm) applies to each diagram in A-D.

The positions of the microdialysis probes in the CeLC and striatum (caudate-putamen) were verified histologically (Figure 11).

## Discussion

The key findings of this study are as follows. Inhibition of PKA or ERK, but not PKC, in the CeLC decreases NMDA receptor-mediated synaptic plasticity in the arthritis pain model but has no effect on basal transmission under normal conditions. PKA and ERK inhibitors administered together do not occlude each other's action but have additive effects, suggesting independent signaling pathways for PKA and ERK. PKA activation by forskolin under normal conditions induces an NMDA receptor-mediated synaptic component that mimics synaptic facilitation observed in the arthritis model. This effect is not blocked by the inhibition of ERK activation, arguing against a role of ERK downstream of PKA. Consequently, inhibitors of PKA and ERK, but not PKC, in the CeLC decrease supraspinally (vocalizations) and spinally (withdrawal reflexes) organized pain behaviors in animals with arthritis but not in normal animals.

The significance of these results is that in the amygdala PKA and ERK, but not PKC, modulate information processing and behavior through separate (not serially arranged) signaling pathways. This is different from pain-related plasticity in the spinal cord [39,40] and from other models of plasticity such as hippocampal long-term potentiation (LTP) [61], where PKA and PKC act in concert to activate ERK. In dorsal horn neurons activation of PKA, PKC, or ERK increased neuronal excitability and inhibited transient potassium (A-type) currents. The effects of PKA and PKC activators were blocked by inhibitors of ERK signaling, suggesting that PKA and PKC act as upstream activators of ERK [39,40]. Spinal PKA and PKC activation has also been implicated in central sensitization [62] and behavioral hypersensitivity [63-65] in different pain models. More recent studies showed ERK activation and antinociceptive effects of ERK inhibition in the spinal cord in several pain models [reviewed by [35]]. The lack of evidence for the involvement of PKC in the present study was somewhat surprising. However it has been pointed out before that "studies on the effects of PKC on NMDA receptors have yielded conflicting results, probably because PKC has multiple effects depending on cell type, sites of action, and variable associations of NMDA receptors with other proteins" [42].

Our data suggest that NMDA receptors are the target of PKA and ERK. NMDA receptors have been shown to function as "upstream" activators of protein kinases. NMDA receptors couple directly [66] or via PKA and PKC [61,67] to ERK activation and are involved in pain-related ERK activation in the spinal dorsal horn [see [35]]. NMDA receptor dependent ERK activation plays an important role in the central sensitization of dorsal horn neurons [68]. However, NMDA receptors are also "downstream" targets of protein kinases. PKA, PKC, and ERK can phosphorylate NMDA receptors to enhance current flow through the receptor and accelerate the kinetics of the ion channel [41-43,69-71]. PKC mediated NMDA receptor phosphorylation removes the magnesium block [72], rendering the channel functional even at normal resting membrane potentials as observed in the present study. Pain-related NMDA receptor phosphorylation of spinothalamic tract (STT) cells in the deep dorsal horn requires both PKC and PKA, whereas phosphorylation in superficial dorsal horn STT cells is due to the action of PKA [73]. The contribution of ERK-mediated NMDA receptor phosphorylation to pain-related neuronal and behavioral changes remains to be determined, but a recent study showed ERK-mediated NMDA receptor phosphorylation by brain-derived neurotrophic factor (BDNF), which can modulate nociceptive transmission in the spinal dorsal horn [41].

The effectiveness of protein kinase inhibitors in the present study suggests tonic NMDA receptor phosphorylation in amygdala neurons in the arthritis pain state. Kinetics of phosphorylation by PKA and ERK are fast (1–5 min) [41,41,43]. PKA can overcome constitutive protein phosphatase activity and rapidly enhance NMDA receptor currents [see [42]]. Blocking phosphorylation with PKA and ERK inhibitors would shift the balance from phosphorylation toward dephosphorylation by constitutively active phosphatases [71]. For example, type I protein phosphatase (PP1) binds to an NMDA receptor-associated protein and decreases current flow through the channel [70]. Striatal enriched tyrosine phosphatase (STEP) is a component of the NMDA receptor complex and can prevent hippocampal LTP without affecting normal synaptic transmission [74]. STEP immunoreactivity is found in cell bodies in several brain areas, including the amygdala [75]. Therefore, the negative regulation of NMDA receptor function by protein kinase inhibitors in the present study can be explained by the relative dominance of constitutively active phosphates.

The mechanisms leading to pain-related PKA and ERK activation in the amygdala remain to be determined. A variety of neuromodulator/neurotransmitter receptors, including metabotropic glutamate receptors that are important for pain-related plasticity in the amygdala [12,16], have been shown to couple to ERK activation via PKA and PKC [61]. Evidence from our previous studies suggests that neuropeptide receptors CGRP1 and CRF1 contribute to pain-related changes in the amygdala through a mechanism that involves PKA activation [10,33]. If PKA and ERK are indeed activated through different mechanisms as the present study may suggest, neuropeptide receptors could activate PKA whereas metabotropic glutamate receptors could couple to ERK activation.

Some methodological aspects need to be considered. The conclusions of this study rely on the selectivity of the protein kinase inhibitors. The role of PKA was determined by using two compounds that inhibit PKA activation through different mechanisms. KT5720 is a widely used selective PKA inhibitor (at nanomolar to low micromolar concentrations) that binds to the catalytic subunits of PKA, causing the displacement of the regulatory subunit and thereby inhibiting the phosphorylating activity of the kinase [50,60]. cAMPS-Rp is a competitive antagonist of cAMP-induced activation of PKA (selective in the low to mid micromolar range) by interacting with cAMP binding sites on the regulatory subunits to prevent cAMP-induced dissociation and activation of the enzyme [51]. Both inhibitors had similar effects. Although these compounds are membrane permeable, we showed that direct intracellular injection of KT5720 had the same effect as perfusion of the slice, confirming an intracellular site of action. U0126 is a well established, membrane-permeable and highly selective inhibitor of ERK activation (at nanomolar to low micromolar concentrations) by directly inhibiting the mitogen-activated protein kinase kinase family members, MEK-1 and MEK-2 [52]. The MEK/ERK-selectivity of U0126 is supported by the fact that the inactive structural analogue U0124 had no effect. PKA and ERK inhibitors had additive effects that were not mimicked by a selective PKC inhibitor (GF109203x (Toullec et al., 1991), further arguing against non-specific effects.

In this study we used protein kinase inhibitors rather than activators, because we sought to determine the role of endogenously activated kinases. Exogenous activation of PKA with forskolin was used to determine the interaction with ERK. We did not test phorbol esters, which are commonly used to activate ERK, because they do so through PKC activation [2,61], which does not appear to be involved in arthritis pain related plasticity in our studies. Therefore, phorbol esters would not mimic the endogenous situation but possibly confound the analysis of ERK function.

Another issue concerns the use of microdialysis for drug application in the behavioral studies. Microdialysis offers several advantages, including continued drug delivery and steady state levels without a volume effect [76]. However, the dose delivered by microdialysis is not known. Based on our previous microdialysis studies of similar-sized non-peptide compounds, we used drug concentrations in the microdialysis fiber that were 100 times higher than the target concentration in the tissue [50,51,53] because of the concentration gradient across the dialysis membrane and diffusion in the tissue[10-14,33]. A "dilution" factor of 100 is further supported by the qualitatively and quantitatively similar effects of drug concentrations applied to the brain slices in the electrophysiological studies and those administered by microdialysis in the behavioral studies.

Finally, it may be surprising that the kinetics of the NMDA component and the compound EPSC were largely similar, whereas data in the literature suggest that NMDA receptors mediate slow EPSCs of relatively long duration [for recent review see [77]]. In addition, NMDA receptor-mediated EPSCs could be recorded at a holding potential of -60 mV, where NMDA receptor channels are normally blocked by magnesium. The NMDA component was isolated pharmacologically with NBQX and bicuculline and was only present in slices from arthritic animals, which is consistent with our previous study [17] that showed similar characteristics of NMDA receptor-mediated synaptic transmission in the amygdala in the arthritis pain model. The results can be explained by the effects of receptor phosphorylation. NMDA receptor phosphorylation relieves the magnesium block and renders the channel functional even at -60 mV [72]. NMDA receptor phosphorylation by PKA or PKC also accelerates the rise and decay times of the ion channel [69,78], which explains the absence of apparent differences in the kinetics of NMDA EPSC and compound EPSC in the present study. However, our finding that an NMDA receptor-mediated component was difficult to detect under normal conditions even at depolarized membrane potentials (Figure 6) may suggest that PKA modulates NMDA receptor function through additional mechanisms such as synaptic targeting [78].

In conclusion, the present study shows that PKA and ERK, but not PKC, are important for pain-related plasticity in the amygdala and for the behavioral consequences of this activity change. PKA and ERK target the NMDA receptor possibly through independent signaling cascades. PKA and ERK render normally "silent" NMDA receptors functional in the arthritis pain model. The independence of PKA and ERK signaling and the lack of PKC effects in this study are different from spinal central sensitization and hippocampal LTP and suggest that the role of protein kinases may be more specific than previously thought.

## Competing interests

The authors declare that they have no competing interests.

## Authors' contributions

YF and JH performed patch-clamp recordings, analyzed electrophysiology data, and provided figures and manuscript drafts. JH, TI, MS, HA, and CR obtained and analyzed behavioral data and provided figures and results in abstract form. TI also helped finalize the manuscript. VN conceptualized the hypothesis, designed and supervised the experiments, directed the data analysis, and finalized the manuscript. All authors read and approved the manuscript.
